# Microglia regulate synaptic development and plasticity

**DOI:** 10.1002/dneu.22814

**Published:** 2021-03-08

**Authors:** Megumi Andoh, Ryuta Koyama

**Affiliations:** ^1^ Laboratory of Chemical Pharmacology, Graduate School of Pharmaceutical Sciences The University of Tokyo Tokyo Japan

**Keywords:** microglia, synapse competition, synapse elimination, synapse engulfment, synapse formation

## Abstract

Synapses are fundamental structures of neural circuits that transmit information between neurons. Thus, the process of neural circuit formation via proper synaptic connections shapes the basis of brain functions and animal behavior. Synapses continuously undergo repeated formation and elimination throughout the lifetime of an organism, reflecting the dynamics of neural circuit function. The structural transformation of synapses has been described mainly in relation to neural activity‐dependent strengthening and weakening of synaptic functions, that is, functional plasticity of synapses. An increasing number of studies have unveiled the roles of microglia, brain‐resident immune cells that survey the brain parenchyma with highly motile processes, in synapse formation and elimination as well as in regulating synaptic function. Over the past 15 years, the molecular mechanisms underlying microglia‐dependent regulation of synaptic plasticity have been thoroughly studied, and researchers have reported that the disruption of microglia‐dependent regulation causes synaptic dysfunction that leads to brain diseases. In this review, we will broadly introduce studies that report the roles of microglia in synaptic plasticity and the possible underlying molecular mechanisms.

## 1 INTRODUCTION

It was shown in the 1970s that in the human cortex, synaptic density increases from birth to early development, then decreases during adolescence and remains nearly constant throughout adulthood (Huttenlocher, 1990). This reduction in synaptic density during development has been called synapse elimination. Removal of synapses via developmental synapse elimination, which results in the pruning of unnecessary synapses and the morphological and functional maturation of the remaining synapses, has been thought to be important for the refinement of neural circuits and thus normal brain function. In fact, compared to nondiseased brains, brains with neurodevelopmental disorders such as autism and schizophrenia (SCZ) have aberrantly increased and decreased synaptic density, respectively (Kolomeets et al., 2005; Tang et al., 2014). Therefore, it has been hypothesized that abnormal synapse elimination underlies neurological disorders (Penzes et al., 2011).

Microglia are glial cells in the brain that function as brain‐resident immune cells by removing cellular debris, dead cells, and pathogens from the brain and producing and releasing inflammatory mediators. As early as in 1968, microglia were suggested to regulate synaptic plasticity in facial nerve transection model through synaptic stripping, i.e., physical displacement of synaptic terminals from the surface of neuronal soma and proximal dendrites (Blinzinger & Kreutzberg, 1968). Further, the complement pathway was possibly involved in the synaptic stripping (Graeber et al, 1988). In recent years, it has also been revealed that microglia play a variety of roles in synaptic plasticity in the normal brain under physiological conditions, as well as in pathological conditions. One such role is developmental synapse elimination via phagocytosis or trogocytosis, that is, synapse engulfment, and the molecules that regulate synapse elimination have been identified. Since microglia contribute to the formation of neural circuits during development, disruption of microglia‐dependent synapse elimination is expected to be involved in the development of neurodevelopmental disorders. Indeed, the association between synapse elimination and neurodevelopmental disorders has been studied in various animal models (Andoh, Shibata, et al., 2019; Filipello et al., 2018; Kim et al., 2017; Zhan et al., 2014). In addition, synapse loss has been observed in neurodegenerative diseases caused by neuronal injury, infection, and aging, and the involvement of microglia‐dependent synapse elimination in these diseases has been thoroughly studied in recent years (Hong et al., 2016; Paolicelli et al., 2017; Vasek et al., 2016; Werneburg et al., 2020). Furthermore, the development and use of genetic techniques that enable time‐ and brain region‐specific microglial removal and regulation of molecular expression have revealed that microglia are also involved in synapse formation during development and learning (Miyamoto et al., 2016; Parkhurst et al., 2013).

**FIGURE 1:**
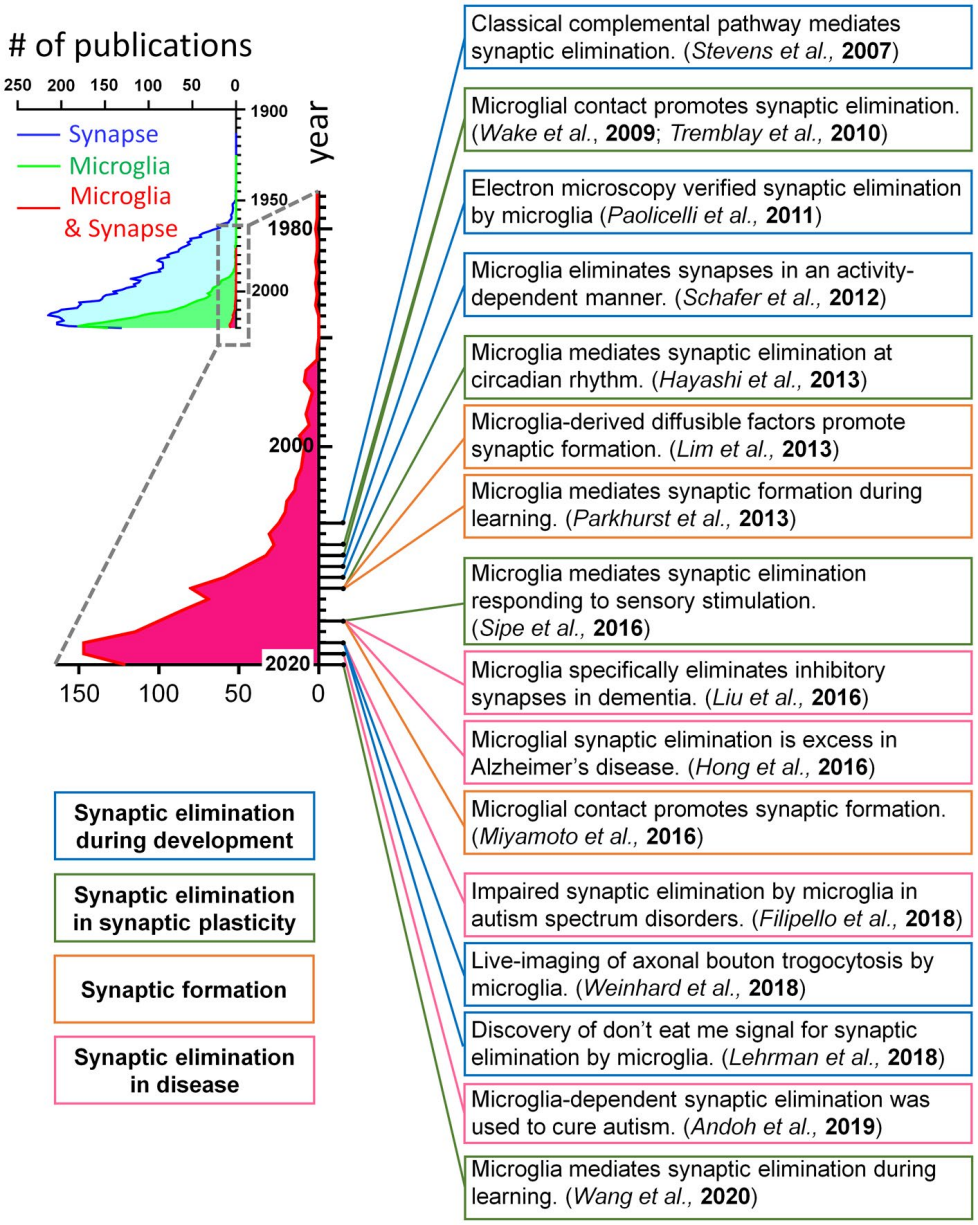
Groundbreaking studies on the microglia–synapse interaction. Some of the essential groundbreaking studies in the history of microglial research are listed in chronological order. These studies help us deeply discuss the mechanisms underlying the regulation of synaptic development and plasticity by microglia. These works improved our comprehension of microglia–synapse interactions, which is reflected by an explosive increase in related papers over the past 15 years. The number of publications was analyzed using PubMed

**FIGURE 2:**
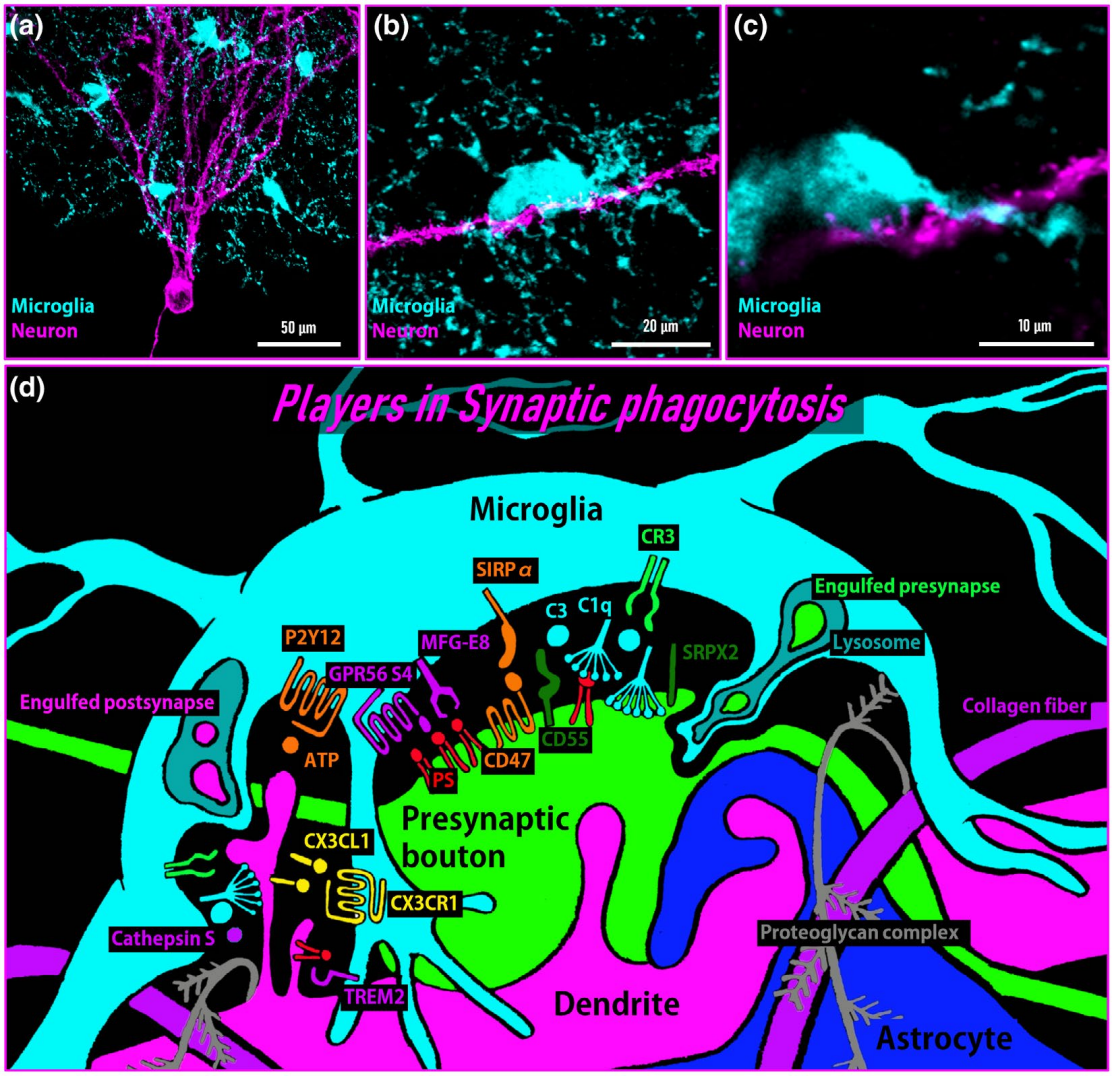
Visualization of the microglia–synapse interaction. (a) Iba1‐immunostained microglia and a GFP‐labeled neuron in the mouse dentate gyrus. (b) A representative image of the microglia–spine interaction. (c) A single plane of the magnified image in b. (d) Illustration of players in synaptic phagocytosis by microglia. It should be noted that the environment surrounding microglial‐synaptic phagocytosis consists of not only microglia and spines but also presynaptic boutons, astrocytes, and extracellular matrix as well as several molecules related to synaptic phagocytosis

In this review, we will describe past studies that have contributed to unveiling the mechanisms by which microglia regulate synaptic plasticity (Figure 1). We will discuss the cellular signaling pathway that has been shown to regulate microglia–synapse interactions and how its disruption affects brain functions. Finally, in addition to the known molecular mechanisms, we will discuss the factors that may regulate microglia‐dependent synaptic plasticity (Figure 2).

## 2 MICROGLIA IN SYNAPSE FORMATION (TABLE 1)

Live in vivo imaging of microglia and spines has suggested that microglial contact with spines modulates synapse formation; in 2016, Miyamoto et al. performed in vivo multiphoton imaging of the sensory cortices of postnatal 8‐ to 10‐day‐old mice, where abundant synaptogenesis occurs (Miyamoto et al., 2016). The rate of filopodium formation was significantly higher in dendritic shafts with microglial contact than in dendritic shafts without microglial contact. Development‐specific removal of microglia in an Iba1‐tTA::tetO‐DTA mouse model resulted in a decreased miniature excitatory postsynaptic current (mEPSC) frequency in sensory cortical pyramidal cells and decreased functional connectivity, which indicates that microglia are required for spine formation. Inhibition of microglial pro‐inflammatory response by minocycline did not change the frequency of microglial contact with dendrites but decreased the rate of filopodium formation, indicating that signal transduction between microglia and dendrites is required for spine formation. Next, Ca^2+^ imaging using GCaMP6m showed that the rate of filopodium formation was higher when microglia were in contact with dendrites than when microglia were not in contact with dendrites and was accompanied by a local increase in Ca^2+^ concentration at the dendritic shaft. Furthermore, minocycline treatment reduced the mRNA expression of Iba1, a Ca^2+^‐binding protein that is often used as a microglial marker. These results suggest that an increased Ca^2+^ concentration in the dendritic shafts due to microglial contact may be important for spine formation.

**TABLE 1:** Synaptic formation

Age	Region	Molecule	Event	Promote or inhibit	Inhibition method	Measurement	Reference
P18–25	Hippocampus	DAP12	Developmental formation	Promote	Mutation	AMPAR/NMDAR ratio	Roumier et al. (2008)
15 DIV	Neuron‐microglia co‐culture	IL‐10‐IL‐10R	Developmental formation	Promote	‒	Density of spine	Lim et al. (2013)
P30, 60	Motor cortex	BDNF‐TrkB	Motor learning	Promote	Microglia‐specific knockout	Live imaging of spine turnover	Parkhurst et al. (2013)
P8–10	Sensory cortex	‒	Developmental formation	Promote	Microglial depletion Minocycline	Live imaging of spine turnover	Miyamoto et al. (2016)
10–12 wo	Olfactory bulb	CX3CR1	Adult neurogenesis	Promote	Knockout	Density of spine	Reshef et al. (2017)
6–7 wo	Hippocampus	CX3CR1	Developmental formation	Promote	Knockout	Functional connectivity Paired pulse ratio	Basilico et al. (2019)

Abbreviations: AMPAR, α‐3‐hydroxy‐5‐methyl‐4‐isoxazole propionic acid receptor; BDNF, brain‐derived neurotrophic factor; CX3CR1, CX3C chemokine receptor 1; DAP12, DNAX‐activating protein of 12 kDa; DIV, day in vitro; NMDAR, *N*‐methyl‐D‐aspartate receptor; P, postnatal day; TrkB, tropomyosin receptor kinase B; wo, week‐old.

In 2017, Reshef et al. also suggested the involvement of microglia in spine formation during adult neurogenesis in the olfactory bulbs; microglial depletion by PLX5562, an inhibitor of colony‐stimulating factor 1 receptor (CSF1R), reduced spine density in adult‐born neurons (Reshef et al., 2017). In vivo two‐photon imaging of microglia in the olfactory bulbs showed an increase in stable spines and a decrease in the proportion of spines that appeared or disappeared in the microglia removal group. This suggests that microglia promote the structural plasticity of spines, which was also reduced in mice with knockout of the fractalkine receptor CX3CR1 (CX3CR1 KO mice) as well as in microglia‐depleted mice. In CX3CR1 KO mice, contact with dendritic shafts was reduced, while contact with dendritic spines was increased; if microglial contact with dendritic shafts is important for spine formation, as observed in Miyamoto et al. (2016), then it is conceivable that CX3CL1‐CX3R1 signaling may regulate microglial contact with dendrites and subsequent spine formation.

In 2019, Basilico et al. suggested the involvement of microglia in presynaptic maturation (Basilico et al., 2019); the authors used acute hippocampal slices to electrophysiologically evaluate the development of synapses that formed between pyramidal neurons in the CA3 and CA1 fields. In CX3CR1 KO mice, the AMPAR/NMDAR ratio did not increase, and the functional connectivity between CA3 and CA1 decreased, which indicated that the synapses failed to mature. In addition, CX3CR1 KO mice had a reduced paired pulse ratio in CA1 pyramidal neurons and a reduced probability of presynaptic release. On the other hand, postsynaptic function was comparable between the control and CX3CR1 KO groups, suggesting that the decline in synaptic function observed in CX3CR1 KO mice was due to changes in the properties of presynapses. Furthermore, CX3CR1 KO mice showed a more activated form of microglia in the hippocampal CA1 field, including hypertrophied cell bodies and decreased numbers of branches on their processes. In addition, the microglial processes of the KO mice showed more linear behavior, although the extension and contraction rate of processes was comparable to that of control mice. These results suggest that changes in microglial process motility may have altered microglial‐synaptic interactions and affected presynaptic maturation.

In addition to physical contact by microglia, microglia‐derived diffusible factors have been shown to regulate synapse formation. In 2008, Roumier et al. used mice deficient in DNAX‐activating protein of 12 kDa (DAP12) function (DAP12^KI^ mice) to examine synapse development. DAP12 is expressed in microglia in the brain, where it forms a complex with triggering receptor expressed on myeloid cells 2 (TREM2), whose downstream signals negatively regulate microglial functions, such as phagocytosis and inflammatory cytokine production. Because DAP12 expression in hippocampal microglia was observed from Embryonic Day 17 to Postnatal Day 3, the authors predicted that DAP12 is involved in the formation of excitatory synapses. In acute hippocampal slices and primary hippocampal neuronal cultures from DAP12^KI^ mice, the AMPAR/NMDAR ratio in CA1 pyramidal cells was increased, which suggests that DAP12 is involved in the synaptic maturation process. In addition, in their previous report (Roumier et al., 2004), immature synaptic phenotypes, such as a high sensitivity of NMDARs to NR2B inhibitors and high AMPAR Ca^2+^ permeability, were confirmed in DAP12^KI^ mice. Furthermore, since the production of cytokines, such as NOS‐2, IL1‐β, and IL‐6, was increased in DAP12^KI^ mice, it is possible that microglia may suppress synaptic maturation via these cytokines.

In 2013, Lim et al. examined the impact of microglia on synapse development using primary rat cortical and hippocampal neuronal cultures (Lim et al., 2013). Neurons cocultured with microglia had a higher density in both presynapses and spines than isolated neuronal cultures. The authors studied cytokines secreted by microglia and found that IL‐10 promoted the formation of both excitatory and inhibitory synapses. In contrast, IL‐1β, IL‐6, IL‐4, and TNF‐α had no effect on synapse formation; IL‐10 was secreted by microglia in a constitutive manner, but the mRNA expression of neuronal IL‐10 receptor subunit α was found to be specific to a period when synapse formation was active (7 DIV in vitro, 3 days of age in vivo). These results suggest a possible mechanism by which the balance between synapse formation and elimination is tilted in favor of elimination by a reduction in IL‐10 receptor expression after synapse formation is induced in immature neurons. Synapse formation in neurons was also promoted by the addition of IL‐10 alone, without coculture with microglia. Given that microglial contact with dendrites may induce synapse formation in some cases (Miyamoto et al., 2016), the overall induction of synapse formation by diffusible factors may serve a different function than the local promotion of synapse formation by contact.

In 2013, Parkhurst et al. examined the relationship between the structural plasticity of synapses and microglia during learning via time‐specific functional manipulation of microglia using CX3CR1‐CreERT2 mice. In vivo two‐photon imaging of spines in the motor cortices showed that both spine formation and removal were reduced in the microglia‐depleted group. When rotarod training was used to induce structural plasticity of spines in the motor cortex, the rate of spine formation after learning was reduced, and motor performance was decreased in the microglia‐depleted group. Furthermore, microglial depletion decreased performance in contextual fear conditioning and novel object recognition tests. In the microglia‐depleted group, the abundance of synapse‐related proteins (VGlut1 and GluN2B) in synaptosomes and the frequency of NMDAR‐ and AMPAR‐dependent mEPSCs decreased. These results suggest that microglia are involved in the formation of functional synapses during learning. Next, the authors focused on a previous study (Coull et al., 2005) showing that microglia‐derived brain‐derived neurotrophic factor (BDNF) promotes the structural plasticity of synapses in a mouse model of neuropathic pain and tested the possibility that BDNF may also be involved in synapse formation during learning. They found that microglia‐specific knockout of BDNF reduced the amount of synapse‐associated proteins and of rate of learning‐dependent spine formation in mice as well as when microglia were depleted; however, it remained unknown what triggers BDNF secretion from microglia. Based on the finding that BDNF release from neurons is promoted in an activity‐dependent manner (Balkowiec & Katz, 2000), it was hypothesized that BDNF may be released from microglia located in the vicinity of highly active synapses. Indeed, considering that neuronal activity‐dependent release of ATP promotes BDNF release from microglia by activating P2X4 receptors in microglia (Khakh & North, 2012) and that ATP induces microglial processes (Davalos et al., 2005), it is possible that neural activity may define the localization of microglia‐dependent synapse formation.

## 3 MICROGLIA IN SYNAPSE ELIMINATION

### 3.1 Synapse elimination under physiological conditions (Table 2)

In vivo two‐photon imaging has revealed that microglia and synapses interact via physical contact. In 2009, Wake et al. found that microglia contacted axonal boutons and dendritic spines in the mouse sensory or visual cortex (Wake et al., 2009). They showed by manipulating neural activity through visual stimulation and thermoregulation that microglial contact with synapses increased with elevated neural activity. Furthermore, the duration of microglial contact with boutons was prolonged after induction of ischemia, and more than half of the contacted boutons disappeared. These studies have motivated neuroscientists to study microglial‐synaptic interactions but have not elucidated the cellular and molecular mechanisms underlying these interactions, and future studies are needed to examine whether microglia may monitor synapses via contact and remove abnormal synapses via phagocytosis.

**TABLE 2:** Synaptic elimination under physiological conditions

Age	Region	Molecule	Event	Promote or inhibit	Inhibition method	Measurement	Reference
P15	Hippocampal CA1	CX3CR1	Developmental elimination	Promote	Knockout	Density of PSD95 and spine	Paolicelli et al. (2011)
P5	LGN	C1q (C3)–CR3	Developmental elimination	Promote	Knockout	Engulfment assay for VGluT2 and axon terminal	Schafer et al. (2012)
8–10 wo	Cortex	Cathepsin S	Sleep	Promote	Knockout	Density of spine	Hayashi et al. (2013)
P28	Visual cortex	P2Y12	Ocular dominance	Promote	Knockout	Engulfment assay for GluR1	Sipe et al. (2016)
P28	Visual cortex	CX3CR1	Ocular dominance	No change	Knockout	Ocular dominance index	Lowery et al. (2017)
	LGN		Developmental elimination	No change		Overlap area of contralateral and ipsilateral input	
P18–20	Hippocampal CA1	TREM2	Developmental elimination	Promote	Knockout	Engulfment assay for PSD95 Density of VGluT1	Filipello et al. (2018)
P5	LGN	CD47‐SIRPα	Developmental elimination	Inhibit	Knockout	Engulfment assay for VGluT2 and axon terminal	Lehrman et al. (2018)
P20, 1–1.5 mo	Hippocampal CA1 Motor cortex	TREM2	Developmental elimination	Inhibit	Knockout	Engulfment assay for PSD95	Jay et al. (2019)
10–12 wo	Hippocampal CA1	C1q (C3)–CR3	Learning	Inhibit	CD55	Engulfment assay for mCherry used to label neurons	Wang et al. (2020)
P5	LGN	PS‐GPR56 S4	Developmental elimination	Promote	Knockout	Engulfment assay for axon terminal	Li et al. (2020)
P10, 21	Hippocampal CA1					Density of colocalization of VGluT2 and Homer1	
P5	LGN	PS	Developmental elimination	Promote	Annexin V	Frequency of mEPSCs of primary hippocampal neuron	Scott‐Hewitt et al. (2020)
P10	Hippocampal CA1						
P10	LGN	SRPX2	Developmental elimination	Inhibit	Knockout	Engulfment assay for axon terminal	Cong et al. (2020)
P60	Somatosensory cortex layer IV						

Abbreviations: C1q, complement component 1q; C3, complement component 3; CD47, cluster of differentiation 47; CR3, complement receptor 3; CX3CR1, CX3C chemokine receptor 1; GPR56 S4, G protein‐coupled receptor 56 isoform 4; LGN, lateral geniculate nucleus; mEPSC, miniature excitatory postsynaptic currents; mo, month old; P, postnatal day; PS, phosphatidylserine; PSD95, postsynaptic density protein 95; SIRPα, signal regulatory protein α; SRPX2, sushi repeat protein X‐linked 2; TREM2, triggering receptor expressed on myeloid cells 2; VGluT1, vesicular glutamate transporter 1; VGluT2, vesicular glutamate transporter 2; wo, week‐old.

In 2010, Tremblay et al. also performed in vivo two‐photon imaging of microglia and dendrites in the visual cortex. When mice were transferred from a dark environment to a light environment to elicit neural activity, the motility of microglial processes increased. Electron microscopy showed that microglial contact with synapses was increased in mice transferred from dark to light environments. Furthermore, live imaging confirmed that the size of spines increased during microglial contact and decreased after contact. Spines with microglial contact had a higher rate of subsequent disappearance than spines without microglial contact, suggesting that microglia are responsible for synapse elimination.

#### 3.1.1 Synapse elimination during brain development

In 2011, Paolicelli et al. investigated the link between synapse elimination and the role of microglia in the hippocampal CA1 field. In postnatal 15‐day‐old mice, microglia were found to incorporate SNAP25, a presynaptic vesicle‐associated protein at the excitatory synapse, and PSD95, which is present in the thickened postsynaptic area (i.e., postsynaptic density). The authors further found that PSD95 and spine density at 15 days of age in CX3CR1 KO mice were increased compared to those in wild‐type mice. Consistent with these results, the excitatory postsynaptic current (EPSC) frequency and amplitude of pyramidal cells were increased in CX3CR1 KO mice, suggesting that microglia are involved in synapse elimination during development through CX3CL1‐CX3CR1 signaling. When pentylenetetrazole (PTZ) treatment induced epileptic seizures, seizure latency was reduced in CX3CR1 KO mice at 17–18 days of age, suggesting that increased synaptic density contributed to the excitability of neural circuits. By contrast, this reduction in latency was not observed in adult mice, suggesting that CX3CR1 KO delays the formation of neural circuits via synapse elimination.

In a paper published around the same time (2012), Schafer et al. presented the first quantitative data on microglial‐synaptic phagocytosis in the retinal–lateral geniculate pathway during development and identified the involved molecular mechanism. The retinal–lateral geniculate pathway has been studied extensively for a long time as a representative example of neural circuits that undergo activity‐dependent synapse elimination. Basically, the lateral geniculate nucleus (LGN) of binocular animals receives overlapping axonal projections and synaptic inputs from retinal ganglion cells (RGCs) of both eyes until early postnatal life. However, during development, ectopic or overformed synapses are removed, and synapses formed in appropriate positions are strengthened, resulting in a distinct regional division of ipsilateral and contralateral axons and synaptic sites. It has long been suggested that ectopically formed synapses are removed in an activity‐dependent manner; Stevens et al. provided the first evidence that this process involves the complement molecule C1q and its downstream component C3, which regulate the classical complement pathway (Stevens et al., 2007). Next, Schafer et al. showed that microglia phagocytose presynapses of RGCs in the dorsal LGN in an activity‐dependent manner during eye segregation. In addition, the number of RGC axons contained in microglia was reduced in mice with knockout of C3 or its receptor, CR3. These results led to the proposal of the following mechanism: first, C1q is released from highly active synapses and tags lower active synapses; then, C1q activates the classical pathway; finally, C3 is fragmented to iC3b. Microglia recognize iC3b via CR3 and phagocytose iC3b‐tagged synapses. Developmental synapse elimination by C1q has also been observed in brain regions other than the LGN: in cortical layer V of C1q KO mice, the bouton density and functional connectivity of excitatory synapses were increased, suggesting that synapse elimination during development was impaired (Chu et al., 2010). In addition, epileptic seizure‐like electroencephalography was observed in C1q KO mice, suggesting that C1q KO induces hyperexcitability of neural circuits by suppressing microglia‐dependent synapse elimination (Chu et al., 2010).

Since these studies, there have been an increasing number of reports of synaptic phagocytosis by microglia. In 2013, Ji et al. examined phagocytosis of synaptic elements by microglia using organotypic slice cultures or primary neuronal cultures prepared from the rodent hippocampus (Ji et al., 2013). First, removal of microglia from hippocampal slice cultures via clodronate treatment increased the EPSC frequency of the neurons, and reintroduction of microglia prevented the increase in EPSC frequency. Similarly, compared to neuronal cultures without microglia, coculture of neurons with microglia reduced the EPSC frequency. Consistent with these electrophysiological results, a decrease in presynaptic and spine densities was also observed upon coculture with microglia. These results suggest that microglia regulate neural circuit formation. Furthermore, in cocultures of neurons and microglia, microglia took up synaptic proteins, indicating that microglia eliminate synapses via phagocytosis. Interestingly, treatment with the microglial repressor MIF (Thr‐Lys‐Pro), which prevents production of TNF‐α and ROS, did not change EPSC frequency, indicating that quiescent microglia, but not active microglia, may be responsible for synapse phagocytosis. However, it should be noted that this study did not exclude the possibility that microglia affect synapse formation.

In recent years, live imaging of synapse phagocytosis by microglia has been achieved thanks to remarkable improvements in culture methods and gene transfer technologies. In 2018, Weinhard et al. performed live imaging of hippocampal slice cultures and captured a series of images of microglial trogocytosis (partial phagocytosis) of presynaptic structures (boutons). The authors claimed in their discussion that microglia did not phagocytose dendritic spines and claimed that synapse phagocytosis by microglia is a presynapse‐specific event, at least under physiological conditions. However, it must be noted that the shape of microglia in tissue culture can differ significantly from that in vivo in terms of cell body size, the complexity of processes (such as length, width, and number of branches), and the expression of steady‐state lysosomes (Kasahara et al., 2016). Therefore, the development of an in vitro experimental system in which an in vivo‐like microglial morphology with highly ramified processes is maintained and more detailed observation of synapse phagocytosis at higher spatial and temporal resolution is possible is needed to directly examine the molecular mechanisms underlying synapse phagocytosis by microglia.

Over the past decade, a variety of molecules have been investigated that regulate synapse elimination by microglia in addition to CX3CL1‐CX3CR1 signaling (Paolicelli et al., 2011) and the C1q‐triggered complement cascade (Schafer et al., 2012), which are described above. One such molecule is TREM2. The innate immune receptor TREM2 is a member of the immunoglobulin superfamily and is expressed in only microglia in the brain. Trem2 mutations have been reported to be involved in the development of various neurodegenerative diseases, such as Nasu‐Hakola disease, Alzheimer’s disease (AD), Parkinson’s disease (PD), and amyotrophic lateral sclerosis (ALS) (Benitez et al., 2013; Cady et al., 2014; Guerreiro et al., 2013; Jonsson et al., 2013; Paloneva et al., 2002). In 2018, Filipello et al. found that synaptic density and mEPSC frequency were increased in the CA1 area of the hippocampus in postnatal 18‐ to 20‐day‐old Trem2 KO mice. They performed phagocytosis assays in vivo and in neuron–microglia cocultures and showed that Trem2 KO reduced synaptic phagocytosis by microglia. By contrast, conflicting results have been reported for the role of Trem2 in synapse removal; in 2019, Jay et al. found that the density of excitatory synapses was reduced in the hippocampal CA1 of postnatal 20‐day‐old and 1‐ to 1.5‐month‐old Trem2 KO mice. They also found that the frequency of mEPSCs in CA1 pyramidal cells was decreased. In CA1, phagocytosis of PSD95 by microglia was not changed, but phagocytosis of PSD95 was increased in the motor cortex. However, these changes observed in Trem2 KO mice were normalized in 4‐month‐old mice. It is possible that this temporary decrease in synaptic density affected brain function, but it remains unknown because behavioral tests were not conducted.

In 2020, studies showed that phosphatidylserine (PS), which is exposed on the surface of apoptotic cells and makes them detectable by phagocytes, regulates synapse elimination during development (Li et al., 2020; Scott‐Hewitt et al., 2020). Scott‐Hewitt et al. found increased presynaptic tagging by PS and increased PS uptake by microglia in the developing LGN and hippocampal CA1. They also showed that in primary hippocampal neuronal cultures, cloaking PS with Annexin V blocked the microglia‐dependent decrease in the frequency of neuronal mEPSCs. These results suggest that PS promotes developmental synapse elimination by microglia. It was also revealed that an adherent G protein‐coupled receptor, GPR56, is involved in the recognition of PS by microglia (Li et al., 2020). In addition to microglia, GPR56 is also expressed by neurons, astrocytes, and oligodendrocytes and regulates myelin formation and cortical lamination during development. Li et al. (2020) showed that microglial GPR56 is important for synapse elimination by using cell type‐specific knockout of GPR56. Interestingly, while GPR56 S4, an alternatively spliced isoform of GPR56, regulates synapse elimination, it does not affect myelin formation or cortical lamination. In addition, it should be noted that depending on the source of the synaptic projection, GPR56 S4 does not regulate synapse elimination, and thus, it is expected that region/projection‐specific mechanisms will be further elucidated.

Importantly, in recent years, the molecular mechanisms that negatively regulate synaptic phagocytosis by microglia have been clarified. The mechanisms that protect necessary synapses from overelimination should guarantee proper synaptic competition during neural circuit formation. In 2018, Lehrman et al. found that the expression level of the “do not eat me” signaling molecule CD47 was increased in RGCs during development (Lehrman et al., 2018). Simultaneously, in the same region, the expression of SIRPα, which is a CD47 receptor, was increased in a microglia‐specific manner. KO of CD47 or SIRPα caused an increase in synaptic phagocytosis by microglia and a decrease in functional synapses, indicating that the CD47‐SIRPα signaling pathway regulates synapse elimination. Furthermore, since CD47 is localized at the axon terminals of more active RGCs, activity‐dependent synapse elimination in the LGN can be coordinately regulated by the C1q‐C3 cascade and CD47. In 2020, Cong et al. identified sushi repeat protein X‐linked 2 (SRPX2) as an inhibitor of microglia‐dependent synapse elimination in the developing LGN and somatosensory cortex layer IV (Cong et al., 2020). SRPX2 is expressed in neurons and has been shown to inhibit the classical complement cascade by binding to C1q and inhibiting the formation of its downstream effector C3.

It has been confirmed that microglia phagocytose synapses in the prefrontal cortex (PFC) and brain stem, in addition to the hippocampus and LGN, during development (Mallya et al., 2019; Milinkeviciute et al., 2019). Therefore, it is possible that microglia‐dependent synapse elimination is a common phenomenon in each brain region. Further studies are awaited to examine whether the molecular mechanisms underlying microglia‐dependent synapse elimination are similar between each brain region.

#### 3.1.2 Synapse elimination by microglia during synaptic modification

Synapse elimination by microglia has been suggested to be involved not only in neural circuit formation during development but also in synaptic modification during adulthood. In 2013, Hayashi et al. examined the diurnal variation of synapses in the mouse cortex and microglia (Hayashi et al., 2013). During sleep, both synaptic activity and spine density were reduced. The authors found that the expression of the microglia‐specific lysosomal cysteine protease cathepsin S showed diurnal variation (with the highest expression when the mice started to wake up) and that knocking out cathepsin S blocked the reduction in synaptic activity and spine density during sleep. These results suggest that cathepsin S secreted by microglia regulates the functional and structural plasticity of synapses. Because cathepsin S is an extracellular matrix protease and the extracellular matrix regulates the maturation and structural plasticity of synapses, it is likely that microglia eliminate synapses during sleep via a mechanism other than phagocytosis.

In 2020, Choudhury et al. showed that microglia also contribute to diurnal variations in synaptic activity and synapse number in the rat PFC (Choudhury et al., 2020). During sleep, microglial activation (cell body hypertrophy and increased CR3 expression) and increased expression of milk fat globulin protein‐E8 (MFG‐E8) facilitate the recognition of “eat me” signals such as complement and PS. Furthermore, the number of C3‐ and MFG‐E8‐bound synapses increases during sleep, and some of these synapses are phagocytosed by microglia. A subsequent study focused on glucocorticoids and noradrenaline, whose expression levels increased during wakefulness, as factors controlling microglia‐dependent synapse elimination (Ishii et al., 2015). Glucocorticoid administration during sleep suppressed the increase of MFG‐E8, CX3CR1, and CD68, and a reduction in noradrenaline by reserpine promoted the expression of C3 and MFG‐E8. These findings suggest that the diurnal variation in synapses is controlled by synapse elimination by microglia.

In 2016, Sipe et al. examined the involvement of microglia in the structural plasticity of synapses in the visual cortex. Neurons in the binocular region of the visual cortex respond to light stimuli to either the left or right eye, but they usually respond more strongly in one of the eyes (ocular dominance). In mice, there is a predominant reaction in the eyes on the opposite side of the visual cortex. However, when one eye is closed during the critical period, the neural circuit is reconstructed so that the open eye becomes dominant. When monocular occlusion was induced in mice during the critical period to induce structural plasticity of synapses due to ocular dominance, the amount of GluR1 phagocytosis by microglia was increased in the region opposite to that showing ocular dominance. On the other hand, in P2Y12 KO mice, structural plasticity of synapses due to eye dominance did not occur, and the increase in GluR1 phagocytosis by microglia was suppressed. These results suggest that microglia may regulate the structural plasticity of synapses by recognizing and phagocytosing synapses whose activity is decreased owing to ocular dominance via P2Y12. In 2017, Lowery et al. examined the involvement of microglial CX3CR1 in the activity‐dependent structural plasticity of synapses in the visual cortex. In 28‐day‐old mice, KO of CX3CR1 did not affect microglial density or motility. In addition, in vivo two‐photon imaging showed no change in the contact time or frequency between microglia and spines. Furthermore, CX3CR1 KO did not show any change in ocular dominance, suggesting that the regulation of structural plasticity of synapses by CX3CR1 varies depending on the developmental stage or brain region.

More recently, in 2020, Wang et al. reported that microglia may be involved in memory loss (Wang et al., 2020). In contextual fear conditioning, microglial depletion by PLX3397 after learning extended the duration of memory retention. The authors examined whether synapse elimination is involved in this process and inhibited complement‐dependent synaptic phagocytosis via expression of CD55, which inhibits C3 activation. CD55 expression prolonged memory retention in mice and reduced phagocytosis of CD55‐expressing neurons by microglia. Further studies are needed to conclude that complement‐dependent synaptic phagocytosis can regulate memory after learning. Again, it should be noted that the authors did not rule out the possibility that synapse formation was reduced due to microglial depletion.

### 3.2 Synapse elimination in diseases (Table 3)

#### 3.2.1 Synapse elimination in neurodevelopmental diseases

Developmental synapse elimination is fundamental for the refinement of neural circuits, and this disruption is thought to lead to abnormal neural circuit functioning, resulting in neurodevelopmental disorders such as autism spectrum disorders (ASDs) (Andoh et al., 2019a, Andoh et al., 2020) and SCZ (Penzes et al., 2011). Therefore, it has been studied whether microglia‐dependent synapse elimination is deficient in animal models of neurodevelopmental disorders or, conversely, whether perturbed synapse elimination by microglia causes neurodevelopmental disorders.

**TABLE 3:** Synaptic elimination in diseases

Disease	Animal model	Age	Region	Molecule	Promote or inhibit	Measurement	Reference
FTLD	Grn knockout	8, 12, 19 mo	Ventral thalamus	C1q(C3)‐CR3	Promote	Area of VGAT	Lui et al. (2016)
AD	J20	3–4 mo	Hippocampal CA1	C1q(C3)‐CR3	Promote	Engulfment assay for Homer	Hong et al. (2016)
Infection	WNV injection	7, 25 dpi	Hippocampal CA3	C1q(C3)‐CR3	Promote	Engulfment assay for synaptophysin	Vasek et al. (2016)
Obesity	High fat diet	Not specified	Hippocampus	‒	Promote	Density of spine Expression levels of synaptic proteins Engulfment assay for synaptosome	Hao et al. (2016)
ASD	atg7 knockout	P12	Sensory cortex	‒	Promote	Engulfment assay for PSD95	Kim et al. (2017)
AD FTLD	TDP‐43 conditional knockout	8 mo	Motor/sensory cortex	‒	Promote	Engulfment assay for PSD95	Paolicelli et al. (2017)
PD	6‐Hydroxydopamine injection	5–7 dpl	Substantia nigra pars reticulata	CR3 CX3CR1 Cathepsin S	Promote	Intensity of synapsin I and PSD95	Aono et al. (2017)
ASD	FMR1 knockout	P1	Hippocampus	‒	Inhibit	Engulfment assay for PSD95 Density of spine	Jawaid et al. (2018)
Injury	TBI	3 dpi	LGN	C1q(C3)‐CR3	Promote	Engulfment assay for axon terminal	Norris et al. (2018)
Obesity	High fat diet	Not specified	Hippocampus	‒	Promote	Density of spine Synaptic inclusion in microglia	Cope et al. (2018)
Injury	TBI	30 dpi	Hippocampus	C1q(C3)‐CR3	Promote	Intensity of PSD95 Engulfment assay for PSD95 injected into brain	Krukwoski et al. (2018)
SCZ	iMG cells and iPSC derived from patients	Not specified	Neuron‐microglia co‐culture	‒	Promote	Engulfment assay for synaptosome and PSD95	Sellgren et al. (2019)
AD	5XFAD:Fgg^γ390−396A^	7–8 mo	Cortex	Fibrinogen‐CR3‐ROS	Promote	Density of spine	Merlini et al. (2019)
ASD	Maternal immune activation	P60	Hippocampal CA3	‒	Inhibit	Engulfment assay for SPO Density of synapse	Andoh et al. (2019c)
MS	EAE	10–12 dpi	LGN	C3‐CR3	Promote	Engulfment assay for VGluT1	Werneburg et al. (2020)
Epilepsy	Kainate‐induced status epilepticus	7, 14, 28 dps	Hippocampus	CX3CR1	Promote	Density of spine	Xie et al. (2020)

Abbreviations: AD, Alzheimer’s disease; ASD, autism spectrum disorders; atg7, autophagy related 7; C1q, complement component 1q; C3, complement component 3; CR3, complement receptor 3; CX3CR1, CX3C chemokine receptor 1; dpi, day post infection or day post injury; dpl, day post lesioning; dps, day post status epilepticus; EAE, experimental autoimmune encephalomyelitis; FMR1, fragile X mental retardation 1; FTLD, frontotemporal lobar degeneration; iMG cells, induced microglia‐like cells; iPSC, induced pluripotent stem cells; LGN, lateral geniculate nucleus; mo, month‐old; MS, multiple sclerosis; P, postnatal day; PD, Parkinson’s disease; PSD95, postsynaptic density protein 95; SCZ, schizophrenia; SPO, synaptoporin; TBI, traumatic brain injury; TDP43, TAR DNA‐binding Protein of 43 kDa; VGAT, vesicular GABA transporter; VGluT1, vesicular glutamate transporter 1; WNV, West Nile virus.

##### ASDs

In 2014, analysis of postmortem brains of ASD patients confirmed the decrease in the spine density of temporal lobe pyramidal cells during development in ASD patients, which resulted in a higher spine density in these patients than in healthy individuals (Tang et al., 2014). Therefore, it has been considered that insufficient synapse elimination causes abnormal neural circuit function in the ASD brain. Trem2 KO mice, in which microglia‐dependent synapse elimination is defective, exhibit ASD‐like behaviors such as stereotype or repetitive behaviors and decreased sociality in adulthood (Filipello et al., 2018). Furthermore, analysis of the postmortem brains of normal subjects and ASD patients confirmed that TREM2 protein levels were reduced in ASD patients.

In 2017, Kim et al. found that mice with knockout of atg7, one of the autophagy‐related genes specific to bone marrow cells, including microglia, exhibited ASD‐like behaviors (Kim et al., 2017). In atg7 KO mice, the spine density and the number of synapse‐related proteins increased in the sensory cortex. Interestingly, the number of PSD95 puncta in microglia was increased. These results imply that synapses taken up by microglia could not be digested as a result of autophagy dysfunction, and further synaptic phagocytosis was inhibited.

FMR1, the causative gene of fragile X syndrome, is located on the X chromosome, and when the number of codon repeats increases, the normal FMR protein is not synthesized, affecting brain development. In 2018, Jawaid et al. reported decreased sociality and persistent behavior in FMR1 KO mice (Jawaid et al., 2018). In adult FMR1 KO mice, the spine density of CA1 pyramidal cells was increased. The authors examined whether the increase in spine density in FMR1 KO mice was the result of the failure of synapse elimination during development, finding that phagocytosis of PSD95 by microglia was significantly decreased in FMR1 KO mice. Depletion of FMR1 caused abnormalities in the functional plasticity of synapses, which may inhibit activity‐dependent synapse elimination.

In 2019, Andoh et al. reported that synapse elimination during development was deficient in the hippocampus of ASD model mice induced by maternal immune activation (Andoh, Shibata, et al., 2019). The authors further determined that the increase in synaptic density was due to the decrease in synaptic phagocytosis by microglia. Furthermore, when the mice were allowed to undergo spontaneous exercise, that is, wheel running, after the development of ASD in adulthood, synaptic phagocytosis by microglia was promoted, and synaptic density and ASD‐like behavior were normalized. Since exercise increased the activity in some dentate gyrus granule cells, it is possible that activity‐dependent synaptic competition induced synaptic phagocytosis by microglia. This work was a groundbreaking study in that it successfully manipulated microglial function in disease via a noninvasive method. In recent years, it has also been suggested that flickering and sound stimuli at a specific frequency (40 Hz) affect microglial morphology and functions, including phagocytosis of Aβ (Adaikkan et al., 2019; Iaccarino et al., 2016; Martorell et al., 2019). These studies suggested that these noninvasive stimuli could modulate synapse elimination by microglia, which prevents the development and deterioration of synaptopathies (Andoh et al., 2020).

##### SCZ

In 2000, a postmortem analysis of the brains of SCZ patients confirmed reduced synaptic density in the cortex and excessive synaptic depletion, and these changes have been suggested as a cause of SCZ (Glantz & Lewis, 2000). In 2019, Sellgren et al. established an in vitro model of microglia‐dependent synapse elimination and examined this possibility (Sellgren et al., 2019). Neurons and synaptosomes derived from SCZ patients were more likely than those derived from healthy controls to be phagocytosed by microglia, and the phagocytic ability of SCZ patient‐derived microglia‐like cells was higher. In addition, the SCZ risk gene C4 was involved in complement attachment to neurons and excess synapse elimination. It was also shown that minocycline suppressed synapse elimination and decreased the risk of developing SCZ.

#### 3.2.2 Synapse elimination in neurodegenerative diseases

**FIGURE 3:**
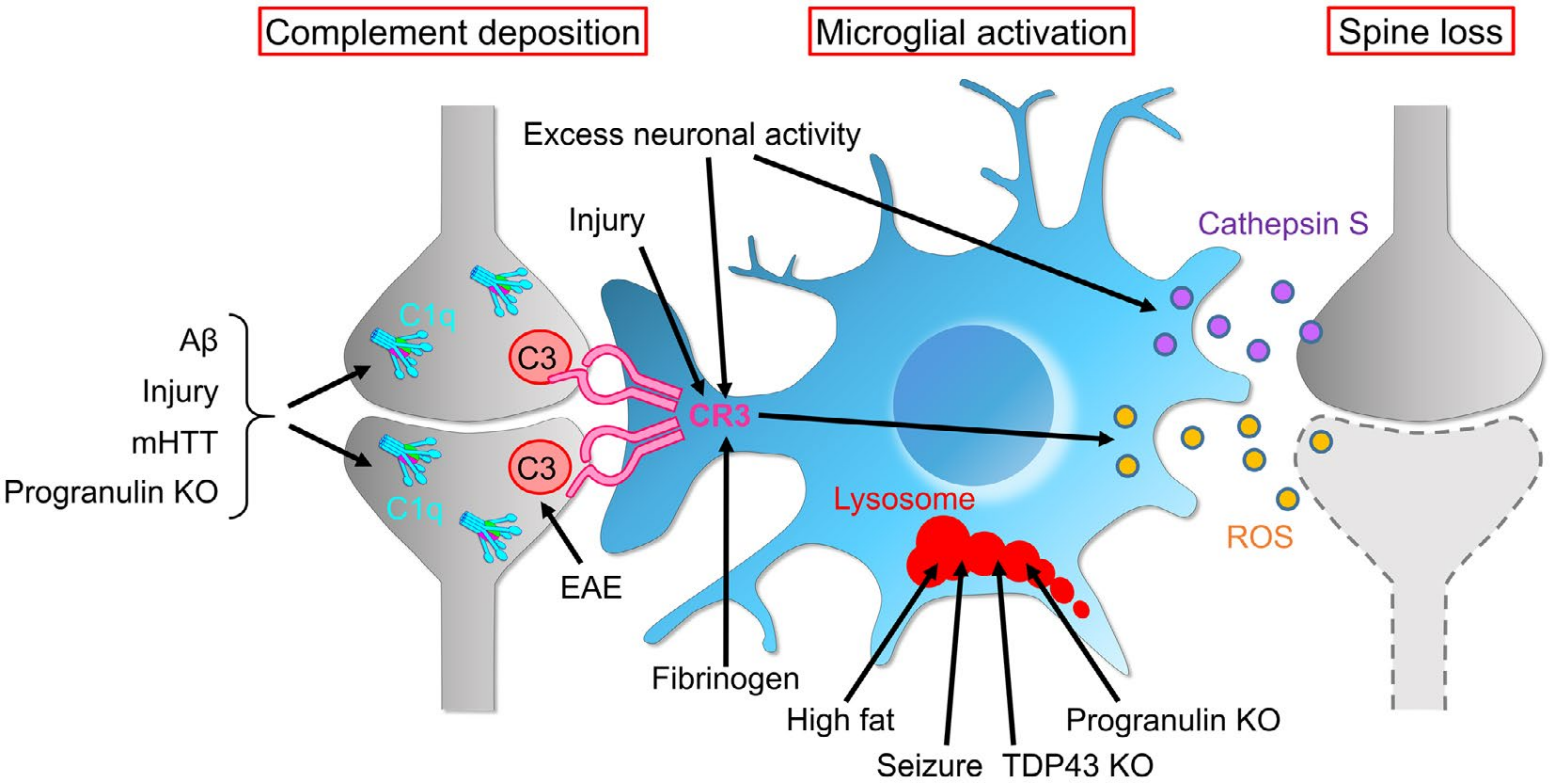
Synapse elimination pathways that are excessively enhanced in neurological disorders. Some neurological disorders induce complement expression and microglial activation, leading to synaptic phagocytosis and spine loss by microglia

Brains with neurodegenerative diseases, such as AD, typically exhibit synaptic dysfunction and synapse loss. Studies using various animal models of neurodegenerative diseases have revealed that protein aggregates, infection, and tissue damage activate microglia to cause excessive synapse elimination (Figure 3). Furthermore, in most of these diseases, abnormal activation of the complement pathway has been reported. Thus, it has been suggested that reactivation of synapse elimination during development is the cause of neurodegenerative diseases (Figure 3).

##### AD

In 2016, Hong et al. found that the accumulation of C1q at postsynapses was increased in J20 mice and mice treated with Aβ oligomers (Hao et al., 2016). The authors further found that phagocytosis of postsynapses by microglia was promoted, resulting in decreased synaptic density. Since Aβ oligomers bind to synapse‐related proteins (Hong et al., 2014; Mucke & Selkoe, 2012), it is possible that Aβ oligomers on the synapse directly bind to C1q and induce microglia‐ and complement‐dependent synapse elimination. Recently, the mechanism by which Aβ increases C1q accumulation in synapses has been elucidated. In 2019, Bie et al. reported that in the hippocampal CA1 region of APP/PS1 mice, Aβ activates mGluR1, which is followed by downstream activation of PP2A and dephosphorylation of FMRP, resulting in local C1q expression at synapses (Bie et al., 2019). It has also been shown that Aβ accumulation may induce oxidative stress and local apoptosis in synapses, which promote PS expression and C1q binding (Györffy et al., 2020). A C1q‐independent mechanism was also proposed in 2019: Merlini et al. reported a mechanism that induces synapse elimination in a manner other than C1q‐dependent synaptic tagging (Merlini et al., 2019). In the cortex of 5XFAD:Fgg^γ390–396A^ mice, invading fibrinogen induced by vascular damage binds to CR3 in microglia and promotes reactive oxygen species (ROS) production in microglia. It was shown that these ROS induce the disappearance of dendritic spines and cause a decline in active avoidance learning performance. However, it remains unclear whether this spine loss was caused by microglial phagocytosis.

##### Frontotemporal lobar degeneration

TAR DNA‐binding protein of 43 kDa (TDP‐43) is a major constituent protein of ubiquitin‐positive inclusions found in neurons and glial cells in the brains of ALS and frontotemporal lobar degeneration (FTLD) patients. TDP‐43 is a ubiquitously expressed nuclear protein and is known to be involved in transcription and splicing. In 2017, it was shown that KO of TDP‐43 in AD model mice promoted microglia‐dependent phagocytosis of not only Aβ but also synaptic proteins (Paolicelli et al., 2017). TDP‐43 KO also increased CD68 expression in microglia and synaptic phagocytosis without Aβ, suggesting that TDP‐43 suppressed microglial phagocytosis. In addition, in 2016, inhibitory synapse‐specific phagocytosis by microglia was reported in mice lacking Grn, which encodes progranulin, an FTLD risk gene (Lui et al., 2016). Progranulin, whose mRNA is mainly expressed in microglia in the brain, is known to suppress the immune response of macrophages. Grn KO increased the expression levels of C1qa and C3 and the phagocytosis of synaptophysin by microglia. Interestingly, excitatory and inhibitory presynaptic tagging by C1q was increased in Grn KO mice, but only inhibitory synapses were reduced. Although the mechanism of inhibitory synapse‐specific phagocytosis has not been clarified, it is possible that the expression and distribution of complement receptors and recognition molecules differ between excitatory and inhibitory neurons.

##### Inflammation

The inflammatory response was first defined by the Roman physician Celsus as responses such as *rubor* (redness), *caloe* (heat), *tumor* (swelling), and *doloe* (pain) (Scott et al., 2004). These inflammatory responses are mediated by cytokines and chemokines released from immune cells stimulated by bacterial and viral infections. Therefore, we reiterate that the definition of true inflammation requires that the three conditions of trigger, mediator, and response be present. However, this definition is mainly proposed based on peripheral inflammation. Thus, it remains an open question whether the definition of true inflammation applies to the brain, which is basically isolated from the peripheral immune system by the blood–brain barrier (BBB) and is considered to be so‐called immune privileged (Forrester et al., 2018).

Since the 1990s, we have frequently encountered the term “neuroinflammation” in published papers. In particular, in recent years, there have been many papers that relate “neuroinflammation” to neurodegenerative diseases such as AD and PD without a clear definition of “neuroinflammation.” However, the nomenclature “neuroinflammation” is often criticized for causing confusion because its definition remains vague and not always consistent from paper to paper and because it is used even when the three conditions of the original definition of inflammation, that is, trigger, mediator, and response, are not all met. For example, many of the studies claiming neuroinflammation do not verify the inflammatory response, which is a condition for inflammation, and refer to changes in glial cell morphology or increases in inflammatory mediator levels as neuroinflammation. The reason for the use of the term neuroinflammation in those studies may be the involvement of molecules and processes common to peripheral inflammation, but increased expression of inflammatory molecules alone should not be considered inflammation in itself if the inflammatory response is not verified (Graeber et al., 2011; Ransohoff, 2016). In such cases, some researchers argue that glial activation or pseudoinflammation is more appropriate rather than neuroinflammation (Graeber et al., 2011). However, it should be noted that the nomenclature “glial activation” also contains ambiguity. We often use the term glial activation to refer to morphological and functional changes in glial cells, such as amoeboid shape, lysosomal hypertrophy, and increased cytokine expression. Therefore, it would be better to accurately and simply describe the details of the changes in the state of glial cells under each experimental condition, rather than consolidating the various changes that glial cells exhibit into a single word, “activation.” Last but not least, it is not clear to us why the prefix “neuro‐” is included in the nomenclature to indicate glial cell state changes in the first place.

A further reason for the confusion in understanding the brain’s immune system is that inflammatory mediators perform apparently different functions from those defined in true inflammation. For example, under physiological conditions, complement molecules regulate synaptic elimination during brain development and learning (Schafer et al., 2012; Stevens et al., 2007; Wang et al., 2020), but few researchers would call these phenomena inflammatory responses. Similarly, in neurodegenerative diseases such as AD and PD, it is obviously prudent not to refer to synaptic or neuronal loss caused by overactivated complement pathways (Hong et al., 2016; Hou et al., 2018) as inflammatory responses. Overall, we conclude that the phenomenon that has been referred to as neuroinflammation is nothing more than a change in the state of neuron–glia interactions mediated by immune‐related molecules without inflammatory responses.

In the following, we will discuss the relationship between synaptic elimination and infection that can cause inflammation, but we will not use the term inflammation unless an inflammatory response is observed in the studies described here.

In 2016, Vasek et al. found that the Barnes maze performance of West Nile virus (WNV)‐infected model mice was impaired, suggesting synaptic dysfunction (Vasek et al., 2016). Immunohistochemical analysis indicated the activation of microglia, such as an increased Iba1‐positive area and increased levels of CD68 expression in the hippocampus of WNV‐infected model mice. WNV infection also increased the expression of complement pathway‐related genes, such as C1qA and C3, in the hippocampus. In addition, in the hippocampal CA3 region, the authors detected decreased synaptic density and increased presynaptic phagocytosis by microglia. Since the decrease in synaptic density was blocked by KO of C3 and C3aR, it was suggested that the complement pathway is activated by WNV infection and that microglia‐dependent synaptic phagocytosis was promoted.

In 2018, Prada et al. examined the association between cytokines released from microglia during infection and synaptic depletion (Prada et al., 2018). Administration of IL‐1β, TNF‐α, and IFN‐γ promoted the release of extracellular vesicles (EVs) from microglia. Among the miRNAs contained in EVs, miR‐146a‐5p reduced the expression of presynaptic and postsynaptic proteins such as synaptotagmin1 and neuroligin1, which contribute to spine formation and maintenance in hippocampal primary neurons. In addition, injection of microglial EVs into the hippocampus of adult mice decreased the frequency and amplitude of mEPSCs and spine density in CA1 pyramidal cells. These results suggest that microglia‐derived EVs promote synapse elimination. It should be noted that it is also possible that microglia‐derived EVs blocked synapse formation.

##### Huntington’s disease

In 2020, Savage et al. reported that in the R6/2 mouse model of Huntington’s disease (HD), CD68 expression in striatal microglia was increased (Savage et al., 2020). Before the onset of motor dysfunction, the number of microglia during phagocytosis, the number of phagosomes in microglia, and the number of synaptic contacts by microglia were increased. Furthermore, in the R6/2 mice, the synaptic density of striatal neurons was decreased. Given that the expression levels of C1qA and C1qB were increased in HD (Kraft et al., 2012), complement‐dependent synaptic phagocytosis may be promoted in HD.

##### Injury

In 2018, Kurowski et al. found that synaptic density in the hippocampus was decreased in a mouse model of traumatic brain injury (Krukowski et al., 2018). Moreover, the expression levels of C1q and C3 were increased in synapses, and the CD11b expression level in microglia was also increased. Consistent with this result, PSD95 phagocytosis by microglia was increased. In the same year (2018), Norris et al. reported that the expression of C1q subunits and CD11b and phagocytosis of axon terminals of retinal neurons by microglia were increased in the LGN in an optic nerve crush mouse model (Norris et al., 2018). These findings indicate that neuronal damage promotes complement‐dependent synaptic phagocytosis by microglia.

##### Multiple sclerosis

In 2020, Werneburg et al. found that phagocytosis of VGluT1 by microglia was increased in the LGN of multiple sclerosis (MS) patients and experimental autoimmune encephalomyelitis (EAE) model mice (Werneburg et al., 2020). Interestingly, in the EAE model mice, synapses were tagged by C3 rather than C1q. In addition, the administration of Crry, which suppresses C3 activation and opsonization, suppressed C3 tagging, synaptic phagocytosis by microglia, and visual function decline. The authors expected the invasion of T cells and fibrinogen into the brain parenchyma due to BBB disruption caused by mechanisms that promote synaptic phagocytosis by microglia in the MS brain. In support of this hypothesis, it has been reported that MS patients show increased T cell infiltration (Lodygin et al., 2019) and that T cells promote microglial‐synaptic phagocytosis (Garber et al., 2019). Furthermore, as mentioned above, fibrinogen leaked from blood vessels has also been shown to promote microglia‐dependent spine removal (Merlini et al., 2019).

##### PD

In 2017, using a rat model of PD generated by 6‐hydroxydopamine (6‐OHDA) administration to the striatum, Aono et al. reported microglial activation, such as cell body hypertrophy, increased CD68 expression and decreased densities of synapsin‐I and PSD95 in the substantia nigra reticulum (SNr) (Aono et al., 2017). On the other hand, phagocytosis of GABAergic synapses by microglia was not promoted. In primary cultures of rat microglia, glutamate treatment increased the expression of synaptic phagocytosis‐related genes (CD11b, CX3CR1, cathepsin S, etc.) and increased the phagocytic activity of microglia. From the above results, in the PD model, glutamate release from the subthalamic nucleus (STN) was elevated, and microglia phagocytosed glutamatergic synapses of the STN neurons formed on substantia nigra/pallidum (SNr/GPi) neurons. In addition, administration of dexamethasone, which is known to protect against dopaminergic neuron damage, decreased the expression levels of synaptic phagocytosis‐related genes in microglia and increased excitatory synaptic proteins, while performance in the rotarod test was decreased. These results suggest that microglia might alleviate PD symptoms by eliminating hyperactive synapses via phagocytosis. Regarding glutamatergic synapse‐specific phagocytosis, the authors discussed the possibility that the expression level of CD47, a don’t eat me signal, was reduced specifically in glutamatergic synapses (no data provided). In addition, it has been shown that GABA may reduce the motility of microglial processes (Fontainhas et al., 2011), which implies that released GABA prevents microglial contact with inhibitory synapses, reducing the opportunity for microglia‐dependent phagocytosis of inhibitory synapses.

##### Epilepsy

It has been proposed that the removal of hyperactive synapses by microglia may occur in the epileptic brain. In 2020, Xie et al. reported that the dendritic spine densities of pyramidal and granule cells were reduced in the hippocampus after status epilepticus (SE) induced by kainic acid (Xie et al., 2020). SE increased the number of M1‐type microglia (CD16/32^+^CD206^‐^) and elevated CD68 expression. On the other hand, SE decreased the expression level of the CD200 receptor, whose ligand is a suppressor of microglial pro‐inflammatory response. From these results, it is possible that pro‐inflammatory microglia may promote synapse elimination after epilepsy. It has also been suggested that the complement pathway is activated in the brains of patients with epilepsy (Wyatt et al., 2017). The expression levels of C1q and iC3b were increased in the cortices of epilepsy patients. Although no comparison was made between control and epileptic patients, it was confirmed that microglia contacted the dendrites of C1q‐attached neurons. Furthermore, in patients with epilepsy, the expression levels of TREM2 and PROS1 (a protein that bridges MerTK and PS and is involved in phagocytosis) were decreased, while the expression of MerTK was increased. These results indicate that in the epileptic brain, activation of the complement pathway may promote synaptic phagocytosis by microglia. Further discussion on the role of microglia in synapse elimination can be found elsewhere (Andoh et al., 2019b).

##### Obesity

Since obesity can cause a decline in memory and learning ability, it is possible that obesity is associated with synaptic dysfunction. Indeed, in the hippocampus of high‐fat diet (HFD)‐fed mice, dendritic spine density and synaptic proteins were decreased in CA1 pyramidal neurons (Cope et al., 2018; Hao et al., 2016). In addition, it has been clarified that hippocampal microglial morphology exhibits an activated form, such as a decrease in branching number, cell body hypertrophy and an increase in the CD68 expression level (Cope et al., 2018; Hao et al., 2016). Furthermore, microglia derived from HFD‐fed mice have an increased capacity for synaptosome phagocytosis (Hao et al., 2016). Interestingly, since a HFD did not affect the amount of *E. coli* phagocytosis by microglia, it is possible that a HFD changes the expression of genes specifically involved in synaptic recognition (Hao et al., 2016).

## 4 REGULATION OF THE SPATIAL SPECIFICITY OF SYNAPSE ELIMINATION

**FIGURE 4:**
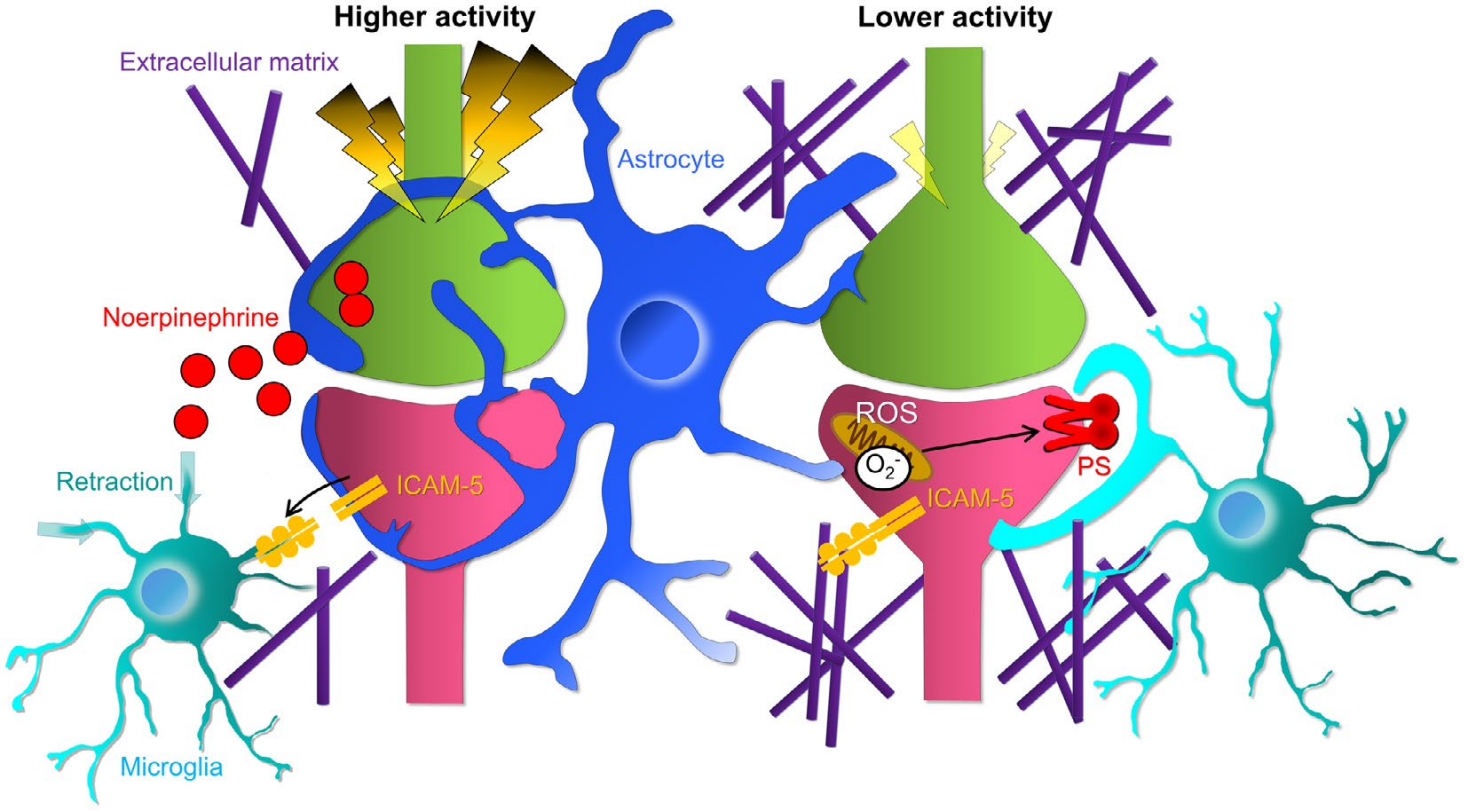
Possible molecular mechanisms by which microglia determine which synapse to phagocytose. Extracellular matrix: Neuronal activity modulates the formation and degradation of extracellular matrix, which affects the ability of microglia to approach synapses. Astrocytes: When neuronal activity is elevated, the area of astrocytes covering synapses is increased, which affects the ability of microglia to approach synapses. Norepinephrine: Norepinephrine is released in an activity‐dependent manner, inducing retraction of microglial processes from synapses. ICAM‐5: ICAM‐5 is released in an activity‐dependent manner, suppressing microglial adhesion to surrounding tissues and phagocytic activity. ROS: ATP demand decreases in synapses with low activity, which promotes ROS production, leading to the expression of PS on synapse surfaces

Why do microglia phagocytose only particular synapses while they come into contact with multiple synapses? In the previous sections, we described the molecular mechanisms regulating synapse elimination that have been revealed in previous studies. It appears that neural activity and synapse tagging by complement are the primary regulatory mechanisms for both synapse elimination during development and synaptic degeneration in the diseased brain. However, not all synapses tagged with C1q and C3 are removed under both physiological (Györffy et al., 2018; Schafer et al., 2012; Stevens et al., 2007) and pathological conditions (Hong et al., 2016; Lui et al., 2016; Werneburg et al., 2020). These results suggest that there may be other mechanisms in addition to the known mechanisms that regulate the selection of prey synapses by microglia. Additionally, although it has been made clear that complement tagging of synapses drives synapse elimination, it remains undetermined whether the number of complement molecules on synapses affects the probability of synapse elimination or whether complement molecules work together with other molecules and cellular dynamics to eliminate synapses. In this chapter, we discuss why microglia choose specific synapses by referring to known and possible factors (Figure 4).

### 4.1 Synapse selection by trogocytosis

In 2018, Weinhard et al. successfully performed live imaging of presynaptic phagocytosis by microglia and showed that after microglial phagocytosis of a part of the bouton, the bouton increased in size (Weinhard et al., 2018). This type of phagocytosis is called trogocytosis. Trogocytosis is found in immune cells, and it has been shown that substances subjected to trogocytosis are used for signal transduction between immune cells (Dance, 2019). Since microglia are able to digest synapses (Kim et al., 2017), microglia can detect the number of synapse‐related proteins and complement molecules during synapse digestion and determine whether to phagocytose more.

### 4.2 Characteristics of synapses that microglia can easily contact

In 2020, Wallace et al. performed in vivo two‐photon imaging of the olfactory bulb and showed that microglia touch mushroom spines more frequently than filopodial spines (Wallace et al., 2020). Because mushroom spines have been shown to be more stable and more active than filopodial spines (Berry & Nedivi, 2017), microglia may contact individual spines in an activity‐dependent manner.

Microglia have a defined territory for individual cells, and their processes rarely overlap with each other in the absence of stimulation, such as neuronal damage (Askew et al., 2017), which may limit the synapses that can be contacted by microglia. In 2018, Iida et al. performed in vivo two‐photon imaging of the sensory cortex, revealing that the closer the synapse was to the microglial cell body, the more frequently microglia contacted the spine and the less stable the spine (Iida et al., 2019). These results suggest that microglia are likely to phagocytose synapses closer to their cell bodies.

### 4.3 Regulation of the extrasynaptic space by the formation and removal of extracellular matrix

The extracellular matrix exists around synapses (Dityatev et al., 2007; Omar et al., 2017), contributing to synapse maturation and structural plasticity (Omar et al., 2017). The extracellular matrix is now regarded as a component of synaptic organization in addition to presynapses and postsynapses, and it is believed that the extracellular matrix controls contact between synapses and glial cells. Perineuronal nets, extracellular matrix structures that serve multiple functions, including modulation of the structural plasticity of synapses, are formed in the visual cortex during the critical period when neuronal plasticity ends (Pizzorusso et al., 2002). Importantly, synaptic phagocytosis by microglia also occurs before the critical period, which suggests that extracellular matrix formation regulates synaptic phagocytosis by microglia.

The relationship between neuronal activity and the extracellular matrix has been verified. For example, in primary cultures of hippocampal neurons, suppression of neuronal activity by TTX treatment reduced the rate of perineuronal net formation in interneurons, which indicates that extracellular matrix formation is promoted by neuronal activity (Dityatev et al., 2007). By contrast, it has also been reported that neuronal activity regulates extracellular matrix degradation. Padamsey et al. performed live imaging of hippocampal slice cultures and primary cultures of hippocampal neurons (Padamsey et al., 2017). They found that Ca^2+^ was released from lysosomes in an action potential‐dependent manner, which promoted exocytosis of the lysosomal protease cathepsin B. cathepsin B activates the extracellular matrix‐degrading enzyme Matrix Metalloproteinase‐9, suggesting that extracellular matrix degradation is activity‐dependent. In addition, in hippocampal neuron primary cultures, neurotrypsin, which is an agrin protease, is secreted from the presynapse in an activity‐dependent manner (Frischknecht et al., 2008). Furthermore, the degradation of agrin by neurotrypsin requires activation of the postsynaptic NMDA receptor (Matsumoto‐Miyai et al., 2009), which suggests that the extracellular matrix around the presynaptic and postsynaptic pair is more easily degraded. These results suggest that neuronal activity may loosen up the extrasynaptic region, promoting glial contact with synapses. However, this hypothesis is inconsistent with the mechanism by which inactive synapses are susceptible to phagocytosis by microglia (Figure 4).

Recently, the relationship between the extracellular matrix and microglia has been studied. Since microglia express some extracellular matrix proteases (Aono et al., 2017; Dwir et al., 2020), it is possible that microglia themselves determine which synapse to contact and degrade the extracellular matrix. In 2020, Nguyen et al. reported that IL‐33 released from neurons promotes phagocytosis of the extracellular matrix (aggrecan) by microglia in the dentate gyrus, which regulates experience‐dependent structural plasticity of synapses (Nguyen et al., 2020). Furthermore, the extracellular matrix components fibronectin and vitronectin cause activation of microglia (increased expression of MHC class I) and increased expression of integrins, including CR3 (Mac‐1) (Milner & Campbell, 2003). Overall, it is possible that the extracellular matrix surrounding the synapse facilitates the detection of C1q by microglia.

The fact that abnormal extracellular matrix formation promotes synaptic degenerative diseases supports the importance of the extracellular matrix in the structural plasticity of synapses. For example, dysplasia of the perineuronal net is common in patients with neurodevelopmental and neurodegenerative diseases, such as SCZ, ASD, and AD (Wen et al., 2018). The perineuronal net is mainly formed around parvalbumin‐positive interneurons, and its failure causes hyperexcitation of neural circuits. Because abnormal synaptic phagocytosis by microglia has been reported in these diseases (Andoh, Shibata, et al., 2019; Hong et al., 2016; Sellgren et al., 2019), it is possible that abnormalities in perineuronal net formation cause dysregulated synaptic phagocytosis by microglia. Particularly in SCZ and AD, synaptic phagocytosis is promoted, which suggests that perineuronal net dysplasia makes it easier for microglia to contact and phagocytose synapses. However, in ASD and fragile X syndrome, in which synaptic phagocytosis by microglia is deficient, dysplasia and accelerated degradation of the perineuronal net have been reported (Sorg et al., 2016; Wen et al., 2018). Therefore, we cannot assert that the perineuronal net necessarily regulates synaptic phagocytosis by microglia.

### 4.4 Astrocytes

Astrocytes form tripartite synapses, which consist of presynapses and postsynapses and astrocytes, and regulate synapse formation, maturation, and activity (Perea et al., 2009). Electron microscopy observations have revealed that astrocyte processes are located close to synapses, raising the possibility that astrocytes regulate microglial contact and phagocytosis of synapses. Moreover, increased neural activity increases the area of astrocytes surrounding synapses and promotes the formation of tripartite synapses (Genoud et al., 2006). These findings suggest the possibility that astrocytes regulate neural activity‐dependent synapse elimination by microglia.

### 4.5 Activity‐dependent factors that could control synapse elimination by microglia

Microglia continuously extend and retract their processes, and this surveillance is considered to be involved in synapse elimination. It has been suggested that dysfunction of the ATP receptor P2Y12 and of the CX3CL1 receptor CX3CR1 alters microglial process motility, affecting synaptic phagocytosis (Paolicelli et al., 2011; Sipe et al., 2016; Tuan, 2019; Zhan et al., 2014). As microglia express various receptors for neurotransmitters and neuronal secretory substances, neuron‐derived molecules other than ATP and CX3CL1 may also regulate microglia–synapse interactions.

The relationship between neuronal activity and microglial surveillance has been thoroughly studied (Dissing‐Olesen et al., 2014; Liu et al., 2019; Madry et al., 2018; Stowell et al., 2019). In 2013, Gyoneva and Trayneils verified the effect of norepinephrine, a typical neurotransmitter, on microglial morphology and process motility (Gyoneva & Trayneils, 2013). Live imaging of acute cortical slices revealed that microglia receive norepinephrine via the β2 adrenergic receptor and retract their processes. In 2019, Stowell et al. performed in vivo two‐photon imaging of the visual cortices of awake mice, showing that norepinephrine inhibited microglial process extension as well as structural plasticity of synapses induced by ocular dominance (Stowell et al., 2019). In addition, a structure called the bulbous tip by which microglia contact synapses (Dissing‐Olesen et al., 2014; Eyo et al., 2014; Li et al., 2012; Wake et al., 2009) was not formed in the presence of norepinephrine. Further studies are needed to examine whether norepinephrine could inhibit synaptic phagocytosis by inhibiting microglial surveillance and contact with synapses.

Neuronal activity regulates the production and transport of various molecules on and within synapses. Intercellular adhesion molecule‐5 (ICAM‐5) is an adhesion molecule that is expressed specifically in excitatory neurons in the telencephalon and is distributed in dendrites and immature spines to regulate the formation and structural plasticity of synapses. It was shown that ICAM‐5 was released from neurons in an activity‐dependent manner (Tian et al., 2007). In addition, in 2017, Paetau et al. showed that ICAM‐5 binds to microglia and inhibits the adhesion of microglia to surrounding tissues (Paetau et al., 2017). Furthermore, ICAM‐5 reduced microglial phagocytic capacity and adhesion to iC3b. From these results, it is possible that highly active synapses may prevent themselves from phagocytosing microglia by releasing ICAM‐5.

ATP is essential for synaptic activity, and approximately 70% of ATP in neurons is consumed by synapses (Harris et al., 2012). Mitochondria are responsible for ATP production, but when ATP production decreases, proton pumping in the electron transfer system decreases. As a result, the electrons leaked into the mitochondrial matrix are directly transferred to oxygen molecules to produce ROS. In fact, suppressing ATP production by suppressing synaptic activity and lowering ATP demand promotes ROS production (Sidlauskaite et al., 2018). In addition, ROS promote the expression of PS on the neuronal membrane. Because microglia recognize PS and phagocytose PS‐tagged synapses (Li et al., 2020; Scott‐Hewitt et al., 2020), PS may function as an “eat me” signal on less active synapses and induce synaptic phagocytosis by microglia. Furthermore, PS has been shown to bind to C1q (Païdassi et al., 2008), and it is possible that PS and C1q work together to promote synaptic phagocytosis by microglia.

Neuronal activity promotes the release of exosomes from neurons. In 2015, Bahrini et al. found that PC12 cell‐derived exosomes increased the phagocytosis of PC12 cell processes by MG6 microglia in their coculture system (Bahrini et al., 2015). Exosome treatment increased C3 expression in MG6 microglia, which suggests that exosomes may regulate synaptic phagocytosis by increasing complement expression. The authors claimed that microglia may prevent hyperexcitation of neural circuits by removing hyperactive synapses. However, it is not clear what is encapsulated in exosomes and what enhances the phagocytic ability of microglia.

## 5 CONCLUSION AND OUTLOOK

In this review, we describe how microglia regulate synaptic plasticity and the structural and functional changes in the brain that result from disrupted microglia–synapse interactions. Although the involvement of various molecules in synaptic plasticity has been revealed, many questions remain to be answered. For example, are the molecular mechanisms identified in previous studies shared by multiple brain regions? Large‐scale gene expression analysis has shown that neurons and microglia have different properties depending on the brain region, and there may be a brain region‐specific mechanism of synaptic plasticity regulation that has not yet been clarified. To further elucidate the interaction between microglia and neurons, particularly synapses, it is necessary to study the involvement of other cell types and the extracellular matrix around synapses. For this purpose, a well‐regulated in vitro system is needed in which cellular structures and mRNA properties reflecting in vivo conditions are reproduced and high‐resolution live imaging and molecular manipulation are possible.

The mechanisms by which microglia phagocytose synapses still need to be further clarified. Recent studies implied that microglia can phagocytose synapses from living neurons, but direct evidence from live imaging and knowledge of the underlying mechanisms are insufficient. It remains unclear whether there is a preference for microglia to eliminate either presynapses or postsynapses, which could be different between healthy and diseased brains or between brain regions. Furthermore, what are the mechanisms by which microglia determine which synapse to phagocytose among adjacent synapses? First, is it critical for the function of a whole neural circuit to determine which among the synapses in close proximity are phagocytosed? To answer this question, we need to examine whether and how neural activity differs between phagocytosed synapses and protected synapses in adjacent locations.

If these questions are answered and the mechanisms of synaptic plasticity by microglia are understood, manipulation of the identified molecules may lead to the treatment of diseases caused by synaptic degeneration. Moreover, if abnormalities of the microglia‐dependent synaptic plasticity can be normalized in advance, it will be possible to prevent disease onset. In recent years, iPSC‐derived microglia that have properties close to those of human microglia have been developed. These tools will be useful for drug discovery research targeting microglia.

Finally, microglia are mesoderm cells and have a different origin from neurons and astrocytes, which are ectoderm cells, leading us to hypothesize that interactions between microglia and other brain cells can be very specific. Why did the brain, which is mainly composed of ectoderm cells, entrust cells of mesoderm origin with the plasticity of synapses, the most essential structures for neurotransmission? Further elucidation of the existence and role of microglia‐specific genes may lead to the discovery of novel mechanisms underlying synaptic plasticity.

## CONFLICT OF INTEREST

The authors declare no conflicts of interests.
